# Mandatory COVID-19 Vaccination for Healthcare Professionals and Its Association With General Vaccination Knowledge: A Nationwide Cross-Sectional Survey in Cyprus

**DOI:** 10.3389/fpubh.2022.897526

**Published:** 2022-05-11

**Authors:** Konstantinos Giannakou, Maria Kyprianidou, Margarita Christofi, Anastasios Kalatzis, Georgia Fakonti

**Affiliations:** Department of Health Sciences, School of Sciences, European University Cyprus, Nicosia, Cyprus

**Keywords:** vaccination, COVID-19, attitudes, healthcare workers, mandatory, obligatory, SARS-CoV-2, Cyprus

## Abstract

COVID-19 compulsory vaccination for healthcare professionals (HCPs) is a sensitive and controversial topic, with different support rates worldwide. Previous studies in Cyprus identified a low COVID-19 vaccination acceptance among HCPs, however, no studies have investigated their perceptions toward mandatory COVID-19 vaccination. This is the first study to investigate the attitudes of HCPs toward mandatory COVID-19 vaccination and its association with general vaccination knowledge. A cross-sectional study was conducted, using an online self-administered, anonymous questionnaire to collect data on sociodemographic and health-related characteristics, trust and satisfaction with the healthcare system, utilization of preventive healthcare services, COVID-19 vaccination information, vaccination knowledge, and attitudes among HCPs toward mandatory COVID-19 vaccination. A total of 504 HCPs participated in the survey, with 34% being in favor of mandatory COVID-19 vaccination. A sufficient vaccination knowledge score was identified among the HCPs, with higher scores being associated with mandatory vaccination support (*p* < 0.001). As age increases by one year, the odds of supporting mandatory vaccination increase by 1.03 units (95% CI: 1.01–1.06). In addition, as the general vaccination knowledge score increases by one unit, the odds of supporting mandatory COVID-19 vaccination increase by 1.55 units (95% CI: 1.33–1.81). Our findings show that about two-thirds of the HCPs in Cyprus were opposed to a mandatory COVID-19 vaccination policy. Older age and general vaccination knowledge were found to be the strongest predictors of mandatory vaccination support. To avoid unforeseen outcomes, mandatory vaccination policies should be implemented with caution and consultation.

## Introduction

Since 2019, a novel coronavirus named severe acute respiratory syndrome coronavirus 2 (SARS-CoV-2), caused the infection and death of millions (1). Although several COVID-19 vaccines have been approved, distributed, and administrated in the general population, a considerable proportion of individuals refuse the COVID-19 vaccination and remain susceptible to infection. To reduce the risk of SARS-CoV-2 infection in the community a plethora of public health measures have been introduced (2). Some of them include the usage of face masks and proof of COVID-19 status/safe pass (evidence of vaccination status, recovery from prior COVID-19 infection, or recent negative test) for traveling abroad and social interaction (3). Mandatory policies may reduce the transmission risk and improve vaccine uptake but cannot tackle the vaccine hesitancy challenge (4).

Vaccine hesitancy refers to refusal or delayed acceptance of available vaccines (5). Interestingly, recent evidence identified healthcare professionals (HCPs) as a hesitant group toward the COVID-19 vaccination worldwide (6). HCPs' occupation includes daily interaction with vulnerable individuals and involves contagious procedures in some facilities. Therefore, some employers in both public and private sectors considering the safety of their staff and patients introduced compulsory COVID-19 vaccination as a condition of deployment and require their employees to be vaccinated before commencing employment, unless exempt (7–9). Existing health and safety procedures in HCPs' profession require the identification of potential hazards, evaluation of the risk, and implementation of strategies to minimize it. Hence, mandating COVID-19 vaccination can be considered an ethically justified duty of HCPs' inherent responsibility for patients' protection. Nevertheless, HCPs may argue that mandating COVID-19 vaccination violates their civil liberties (10).

Mandating vaccination for HCPs is not a new phenomenon (11). Vaccination against seasonal influenza and hepatitis B is a necessity for some HCPs (12). Regarding mandating COVID-19 vaccination for HCPs, Italy was the first country to introduce it with a large proportion of occupational physicians and public health professionals being positive toward the policy (13, 14). In addition, the vast majority of Mongolian HCPs' are in favor of compulsory COVID-19 vaccination (15). Different determinants influence HCPs' attitudes toward mandating COVID-19 vaccination including their vaccination knowledge (16). A recent study in the U.S among medical students revealed a link between vaccination knowledge and attitudes toward mandatory COVID-19 vaccination for patients (17). Furthermore, recent evidence suggests that HCPs with low educational levels are opposed to mandatory COVID-19 vaccination for HCPs (18).

Vaccination of HCPs is not mandatory in Cyprus, (19) however, specific vaccinations are recommended including the vaccination against COVID-19. A low COVID-19 vaccination acceptance rate among HCPs in Cyprus was recently observed, (20, 21) with vaccine safety and efficacy concerns being the main reason for vaccine refusal (20, 22, 23). In addition, a previous study demonstrated that general vaccination knowledge is associated with COVID-19 vaccination acceptance among nurses and midwives in Cyprus (24). Given the low COVID-19 acceptance rate and a potential universal policy toward HCPs' compulsory vaccination, we conducted a survey to elucidate HCPs' perspectives on mandatory COVID-19 vaccination. No studies have previously investigated this topic in Cyprus; therefore, we aim to provide novel insights into factors that affect their attitudes including the role of vaccination knowledge.

## Materials and Methods

### Study Design, Participants, and Data Collection

This study was reported following the Strengthening the Reporting of Observational Studies in Epidemiology (25). An online cross-sectional questionnaire-based survey was performed between 15th of November 2021 and 7th January 2022, involving Greek-Cypriot HCPs working in either public or private service provision, aged 18 years old and above, and living in the five government-controlled municipalities of the Republic of Cyprus (Nicosia, Limassol, Larnaca, Paphos, and Ammochostos). The online questionnaire was administered using Google Forms and dispersed using instant messaging apps (e.g., WhatsApp, Viber), social media platforms (e.g., Facebook, Instagram), and social networking sites (e.g., LinkedIn). Due to the quarantine restrictions resulting from the ongoing COVID-19 pandemic, a nonprobability convenience sampling approach was used to recruit participants. The required sample size to estimate the percentage of HCPs who support mandatory COVID-19 vaccination using a 95% confidence interval (CI) with a precision of 5%, assuming a true percentage of 35–95% was *n* = 139–350.

### Study Instrument

The online questionnaire included 47 open-ended and closed-ended questions in the Greek language about sociodemographic characteristics (e.g., age, gender, educational level, marital status, annual income), health-related status (e.g., presence of chronic diseases), information about their trust and attitudes toward healthcare system, satisfaction with it, as well as use of preventive healthcare services, information about COVID-19 vaccination (e.g., vaccination status, number of doses, type of COVID-19 vaccine, intention to receive another dose if requested etc.), sources of vaccine-related information (e.g., internet/social media, TV/newspapers/radio, scientific journals, personal doctor, colleagues/friends/family etc.), information about the reasons for vaccination refusal (e.g., fear of adverse side effects, expedited development and approval of the vaccine, concerns about getting infected from the vaccine etc.), participants' general vaccine knowledge, and attitudes toward mandatory COVID-19 vaccination. Questions about personal freedoms, human rights, and ethical aspects of mandatory COVID-19 vaccination were used, to reflect the overall attitudes of participants toward such policy. The questionnaire was developed by our research team, based on our previous research experience and extensive literature search (26–32). Validity assessment was done in a pilot study of 50 participants before to the actual study. To achieve a high level of face validity we assess the clarity and application of all survey items, as well as to address wording issues. Specific words were eliminated or replaced based on participants' suggestions to heighten participants' understanding. Internal consistency of the study instrument was assessed with Cronbach's alpha coefficient ranged between 0.63 and 0.96, suggesting acceptable internal consistency. The pilot sample was not included in the study sample.

### Ethics Approval

This study was carried out following the Helsinki Declaration guidelines and was approved by the Cyprus National Bioethics Committee (CNBC) (EEBK E⊓ 2021.01.219). Participation was anonymous, and all participants were informed about study purpose and objectives before taking part. Participants gave their consent before completing the questionnaire by answering a “Yes/No” question on a mandatory electronic form.

### Statistical Analysis

The Shapiro-Wilk normality test was used to examine the normality of the continuous variables. Participants' characteristics are presented as mean ± standard deviation (SD) for continuous measures with normal distribution and absolute (*n*) and relative (%) frequencies for categorical variables. To assess the association between mandatory COVID-19 vaccination and the categorical characteristics, the chi-square test of independence was used. The Student's *t*-test was used for the comparison of mandatory COVID-19 vaccination and continuous baseline participant characteristics with normal distribution.

Participants' vaccination knowledge was measured using a 12-item scale. A vaccination knowledge score was created for each participant by scoring the individual knowledge question items, giving a score of 1 for each question correctly answered and 0 for each question answered incorrectly or in “I do not know” responses. The knowledge score was calculated by adding the points of each of the 12 knowledge items (maximum score 12). The tertiles of vaccination knowledge score were defined as follows: low vaccination knowledge (score ≤ 8), moderate vaccination knowledge (score 8.1–9), and high vaccination knowledge (score ≥ 10). Higher scores indicate a higher vaccination knowledge.

Hierarchical logistic regression analysis was used to examine the association between sociodemographic characteristics (Model 1) and vaccination knowledge score (Model 2) on mandatory vaccination support (Yes vs. No). Firstly, we added the sociodemographic characteristics as independent variables in a model including mandatory vaccination support as the dependent variable. Then, we added the vaccination knowledge score. Radar graphs were constructed to present the reasons for vaccination and the reasons for hesitating to get vaccinated against COVID-19. All statistical tests performed were two-sided with the statistical significance level set at α = 0.05. Statistical analysis was conducted using STATA 14.0 (Stata Corp, College Station, TX, USA) and Microsoft Excel 2013.

## Results

### Participants' Characteristics

There was a total of 504 HCPs participated in the study among whom 223 (48%) were nursing staff, 76 (16.3%) were pharmacists, 73 (15.7%) were physicians, 62 (13.3%) were other Non-medical professionals (i.e., laboratory workers, nutritionists, occupational therapists, psychologists, radiologists, speech and language therapists, care assistants, and administrative personnel) and 31 (6.7%) were physiotherapists (Table 1). The mean age of the respondents was 36.7 years old (*SD* = 9.6). The majority of HCPs were female (*n* = 320, 63.5%) and residents of the capital of Cyprus, Nicosia (*n* = 232, 46.0%). More information about the sociodemographic characteristics is presented in Table 1.

**Table 1:** Sociodemographic characteristics of participants, overall and by mandatory vaccination support.

**Sociodemographic characteristics**	**Overall** **(*N* = 504)**	**Mandatory COVID-19 vaccination**		
		**No ** **(*N* = 328)**	**Yes** **(*N* = 172)**	***p*-value**
**Gender [N^a^ (%)]**				
Female	320 (63.5)	204 (64.3)	113 (35.7)	0.440^h^
Male	184 (36.5)	124 (67.8)	59 (32.2)	
**Mean Age** (SD)	36.7 ± 9.6	34.9 ± 8.9	40.1 ± 10.1	**<0.001** ^h^
**Geographical area [N**^a^ **(%)]**				
Nicosia	232 (46.0)	147 (63.4)	85 (36.6)	**0.001** ^g^
Limassol	98 (19.5)	74 (76.3)	23 (23.7)	
Larnaca	85 (16.9)	45 (54.9)	37 (45.1)	
Paphos	38 (7.5)	20 (52.6)	18 (47.4)	
Ammochostos	51 (10.1)	42 (82.3)	9 (17.7)	
**Marital status [N^b^ (%)]**				
Married/In cohabitation	364 (73.5)	226 (62.4)	136 (37.6)	**<0.001** ^g^
Unmarried	104 (21.0)	84 (80.8)	20 (19.2)	
Divorced/separated/widowed	27 (5.5)	12 (44.4)	15 (55.6)	
**Underage children living in the household [N^a^ (%)]**				
No	236 (46.8)	167 (71.1)	68 (28.9)	**0.015** ^g^
Yes	268 (53.2)	161 (60.7)	104 (39.3)	
**Education [N^c^ (%)]**				
Undergraduate education	251 (50.5)	174 (70.2)	74 (29.8)	**0.025** ^g^
Postgraduate education	246 (49.5)	149 (60.6)	97 (39.4)	
**Annual income [N^d^ (%)]**				
Low (≤ €6,500)	5 (1.0)	3 (60.0)	2 (40.0)	**<0.001** ^g^
Moderate (€6,500–19,500)	159 (32.2)	128 (81.0)	30 (19.0)	
High (>€19,500)	330 (66.8)	190 (57.9)	138 (42.1)	
**Healthcare profesion [N^e^ (%)]**				
Physician	73 (15.7)	34 (46.6)	39 (53.4)	**0.002** ^g^
Nursing staff	223 (48.0)	154 (70.0)	66 (30.0)	
Pharmacists	76 (16.3)	49 (64.5)	27 (35.5)	
Physiotherapists	31 (6.7)	24 (77.4)	7 (22.6)	
Other Non-medical professionals^f^	62 (13.3)	35 (56.4)	27 (43.6)	

*SD, standard deviation.*

a *N = 504.*

b *N = 495.*

c *N = 497.*

d *N = 494.*

e *N = 465.*

f *Other Non-medical professionals included laboratory workers (12), nutritionists (16), occupational therapists (4), psychologists (6), radiologists (4), speech and language therapists (14), care assistants (1), administrative personnel (5).*

g *Differences between mandatory COVID-19 vaccination groups were tested using chi^2^ test.*

h *Differences between mandatory COVID-19 vaccination groups were tested using t-test; Bold values indicate statistically significant associations*.

### HCPs' Characteristics by Mandatory COVID-19 Vaccination

Among the HCPs, 172 (34.4%) were in favor of mandatory COVID-19 vaccination, and 328 (65.6%) were opposed to such a policy. The mean age of those who did not support the mandatory vaccination was 34.9 ± 8.9 years, and for those who support it was 40.1 ± 10.1 years (*p* < 0.001) (Table 1). Most of the residents of all geographical areas were against the mandatory COVID-19 vaccination (*p* = 0.001), while the majority of supporters were physicians (*n* = 39, 53.4%) and other Non-medical professionals (*n* = 27, 43.6%) (*p* = 0.002) (Table 1).

Although we did not find a statistically significant association between gender and HCPs' perspectives toward mandatory COVID-19 vaccination (*p* = 0.440), we identify statistically significant associations among other sociodemographic characteristics that are presented in Table 1. Among those, the largest differences were observed in unmarried individuals (80.8% vs. 19.2%, for no and yes, respectively) (*p* < 0.001), and those who completed an undergraduate education (70.2% vs. 29.8%, for no and yes, respectively) (*p* = 0.025).

Information about HCPs' health status and their attitudes toward healthcare services was retrieved through the survey, with around 20% of HCPs reporting at least one chronic disease. Most of the participants declared the moderate usage of preventive healthcare services (*n* = 183, 36.6%) and had a strong trust in the official guidelines and recommendations of the national healthcare authorities (*n* = 179, 35.7%). Approximately half of the HCPs were moderately satisfied with the healthcare system (*n* = 12, 85.7%), and they follow doctor's instructions very often (*n* = 67, 47.2%). In addition, most of the participants believe that the vaccine helped them to prevent COVID-19 disease a lot (*n* = 144, 38.0%) (Supplementary Table 1). Statistically significant differences between trust in official guidelines and recommendations by the national healthcare authorities, satisfaction with the healthcare system, following doctor's instructions/medical adherence, and mandatory vaccination support groups were identified (*p* < 0.001). Specifically, the largest percentage of participants who support the mandatory COVID-19 vaccination have a very strong trust in the official guidelines and recommendations of the national healthcare authorities (*n* = 59, 77.6%), they are extremely satisfied with the healthcare system (*n* = 12, 85.7%), and they follow doctor's instructions very often (*n* = 67, 47.2%). In addition, most of the participants believe that the vaccine helped them to prevent COVID-19 disease a lot (*n* = 144, 38.0%) (Supplementary Table 1).

### COVID-19 Vaccination Status of HCPs and Their Attitudes Toward Mandatory COVID-19 Vaccination

A considerably high proportion of HCPs were vaccinated against COVID-19 (*n* = 350, 70.4%), with two doses (*n* = 179, 50.7%) of the Pfizer vaccine (*n* = 236, 66.7%) (Supplementary Table 2). The primary reasons for vaccination were to protect themselves (29.3%), their families (28.5%), and others (26.0%) (Supplementary Figure 1), while common reasons for vaccine refusal were the expedited development and approval of the vaccine (26.5%), fear of adverse side effects/safety concerns (25.0%), and preference for natural immunity (17.7%) (Supplementary Figure 2). HCPs retrieved information about the COVID-19 vaccination from scientific journals (24.8%), internet/social media (24.7%), and TV/Newspapers/Radio (14.5%) (Supplementary Figure 3). In addition, we found statistically significant associations between HCPs' COVID-19 vaccination status and mandatory vaccination support groups, which are presented in Supplementary Table 2.

Regarding HCPs' attitudes toward mandatory COVID-19 vaccination, many participants strongly disagree with COVID-19 vaccination mandate (*n* = 200, 39.8%), and that mandatory disposal of COVID-19 vaccines is ethically and scientifically justified (*n* = 160, 31.8%) (Table 2). Additionally, most of the respondents strongly disagree that mandatory COVID-19 vaccination is a policy that will reinforce their understanding that (i) the COVID-19 vaccine is necessary (*n* = 161, 32.3%), (ii) vaccine side effects are rare (*n* = 160, 32.2%), (iii) the vaccine has been studied well (*n* = 163, 32.6%). Approximately one-third of participants strongly agree that mandatory COVID-19 vaccination of HCPs is a policy directed against individual freedoms (*n* = 170, 33.9%) and violates human rights (*n* = 184, 36.7%). Most of the participants strongly agree that COVID-19 vaccination should be mandatory for HCPs (e.g., doctors, nurses, etc.) (*n* = 160, 31.8%). Interestingly, none of the HCPs who disagree or strongly disagree that COVID-19 vaccination should be mandatory for HCPs, support mandatory vaccination, while the corresponding percentages among those who neither agree nor disagree, agree, and strongly agree with that attitude were 10.3, 34.9, and 85.0%, respectively (*p* < 0.001). Finally, we observed statistically significant associations for all the attitudes toward mandatory COVID-19 vaccination among mandatory vaccination support groups (*p* < 0.001) (Table 2).

**Table 2:** Participants' attitudes toward mandatory COVID-19 vaccination, overall and by mandatory vaccination support.

**Mandatory COVID-19 vaccination's attitudes**	**Overall** **(*N* = 504)**	**Mandatory COVID-19 vaccination**		
		**No** **(*N* = 328)**	**Yes** **(*N* = 172)**	***p*-value**
**Extent of support of COVID-19 vaccination mandate [N^a^ (%)]**				
Strongly disagree	200 (39.8)	200 (100.0)	0 (0.0)	**<0.001** ^e^
Disagree	61 (12.2)	60 (100.0)	0 (0.0)	
Neither agree nor disagree	71 (14.1)	65 (94.3)	4 (5.7)	
Agree	76 (15.1)	2 (2.6)	74 (97.4)	
Strongly agree	94 (18.8)	0 (0.0)	94 (100.0)	
**Mandatory COVID-19 vaccination is ethically and scientifically justified [N^b^ (%)]**				
Strongly disagree	160 (31.8)	160 (100.0)	0 (0.0)	**<0.001** ^e^
Disagree	62 (12.3)	61 (100.0)	0 (0.0)	
Neither agree nor disagree	101 (20.1)	78 (78.0)	22 (22.0)	
Agree	105 (20.9)	26 (24.8)	79 (75.2)	
Strongly agree	75 (14.9)	3 (4.0)	71 (96.0)	
**Mandatory COVID-19 vaccination is a policy directed against individual freedoms [N^a^ (%)]**				
Strongly disagree	80 (15.9)	6 (7.6)	73 (92.4)	**<0.001** ^e^
Disagree	84 (16.8)	23 (27.4)	61 (72.6)	
Neither agree nor disagree	80 (15.9)	48 (60.8)	31 (39.2)	
Agree	88 (17.5)	81 (93.1)	6 (6.9)	
Strongly agree	170 (33.9)	169 (99.4)	1 (0.6)	
**Mandatory COVID-19 vaccination violates human rights [N^a^ (%)]**				
Strongly disagree	74 (14.7)	2 (2.7)	72 (97.3)	**<0.001** ^e^
Disagree	97 (19.3)	22 (22.9)	74 (77.1)	
Neither agree nor disagree	66 (13.2)	45 (69.2)	20 (30.8)	
Agree	81 (16.1)	74 (92.5)	6 (7.5)	
Strongly agree	184 (36.7)	184 (100.0)	0 (0.0)	
**Mandatory COVID-19 vaccination is a policy that will reinforce my understanding that the COVID-19 vaccine is necessary [N^c^ (%)]**				
Strongly disagree	161 (32.3)	155 (96.3)	6 (3.7)	**<0.001** ^e^
Disagree	101 (20.2)	77 (77.0)	23 (23.0)	
Neither agree nor disagree	110 (22.1)	60 (55.0)	49 (45.0)	
Agree	96 (19.2)	33 (34.7)	62 (65.3)	
Strongly agree	31 (6.2)	2 (6.4)	29 (93.6)	
**Mandatory COVID-19 vaccination is a policy that reinforces my perception that vaccine side effects are rare [N^c^ (%)]**				
Strongly disagree	160 (32.2)	151 (94.4)	9 (5.6)	**<0.001** ^e^
Disagree	118 (23.6)	82 (69.5)	36 (30.5)	
Neither agree nor disagree	118 (23.6)	61 (52.1)	56 (47.9)	
Agree	79 (15.8)	30 (38.5)	48 (61.5)	
Strongly agree	24 (4.8)	1 (4.3)	22 (95.7)	
**Mandatory COVID-19 vaccination is a policy that will reinforce my perception that the vaccine has been studied well [N^d^ (%)]**				
Strongly disagree	163 (32.6)	160 (98.2)	3 (1.8)	**<0.001** ^e^
Disagree	93 (18.6)	71 (77.2)	21 (22.8)	
Neither agree nor disagree	114 (22.8)	60 (53.1)	53 (46.9)	
Agree	100 (20.0)	32 (32.0)	68 (68.0)	
Strongly agree	30 (6.0)	2 (6.9)	27 (93.1)	
**COVID-19 vaccination should be mandatory for healthcare professionals (e.g., doctors, nurses, etc.) [N^b^ (%)]**				
Strongly disagree	144 (28.6)	144 (100.0)	0 (0.0)	**<0.001** ^e^
Disagree	52 (10.4)	52 (100.0)	0 (0.0)	
Neither agree nor disagree	60 (11.9)	52 (89.7)	6 (10.3)	
Agree	87 (17.3)	56 (65.1)	30 (34.9)	
Strongly agree	160 (31.8)	24 (15.0)	136 (85.0)	

a *N = 502.*

b *N = 503.*

c *N = 499.*

d *N = 500.*

e *Differences between mandatory COVID-19 vaccination groups were tested using chi^2^ test; Bold values indicate statistically significant associations*.

### General Vaccination Knowledge and Mandatory COVID-19 Vaccination

The mean vaccination knowledge score was 8.6 which indicates a sufficient vaccination knowledge (Table 3). We found a statistically significant difference in the mean vaccination knowledge score among mandatory COVID-19 vaccination groups (*p* < 0.001). Specifically, we reported a higher mean vaccination knowledge score among those who support mandatory COVID-19 vaccination (9.5) compared to those who did not support mandatory COVID-19 vaccination (8.1). Also, we found a statistically significant association between vaccination knowledge level and mandatory COVID-19 vaccination groups (*p* < 0.001). Most of the participants who support mandatory COVID-19 vaccination were among those with a high vaccination knowledge level (*n* = 101, 52.6%), followed by those with a moderate vaccination knowledge level (*n* = 33, 29.7%), and those with a low vaccination knowledge level (*n* = 38, 19.3%). More information regarding vaccination knowledge items overall and by mandatory vaccination support are presented in Supplementary Table 3.

**Table 3:** Participants' general vaccination knowledge, overall and by mandatory vaccination support.

**Vaccination knowledge**	**Overall** **(*N* = 504)**	**Mandatory COVID-19 vaccination**		
		**No** **(*N* = 328)**	**Yes** **(*N* = 172)**	***p*-value**
**Mean knowledge score**^a^ (SD)	8.6 ± 2.1	8.1 ± 2.1	9.5 ± 1.5	**<0.001** ^c^
**Knowledge level [N^b^ (%)]**				
Low (score ≤ 8)	198 (39.3)	159 (80.7)	38 (19.3)	**<0.001** ^d^
Moderate (score 8.1–9)	112 (22.2)	78 (70.3)	33 (29.7)	
High (score ≥ 10)	194 (38.5)	91 (47.4)	101 (52.6)	

*SD, standard deviation.*

a *Range of knowledge score 0–12.*

b *N = 504.*

c *Differences between mandatory COVID-19 vaccination groups were tested using t-test.*

d *Differences between mandatory COVID-19 vaccination groups were tested using chi^2^ test; Bold values indicate statistically significant associations*.

### Determinants of Mandatory Vaccination Support and Vaccination Knowledge Score

Hierarchical logistic regression models for sociodemographic and vaccination knowledge scores on mandatory vaccination support were applied (Table 4). Firstly, we applied a model adding various sociodemographic characteristics (Model 1). We found that increased age was associated with a higher probability of supporting the COVID-19 mandatory vaccination (OR: 1.03, 95% CI: 1.01–1.06). Moreover, we revealed that nursing staff had 49% (95% CI: 0.28–0.93) lower probability of supporting mandatory vaccination compared to physicians. When we added vaccination knowledge score in the model (Model 2), we found that only the association for age (OR: 1.03, 95% CI: 1.01–1.06) with COVID-19 mandatory vaccination remained statistically significant. Divorced/separated/widowed individuals had 3.19 times higher probability of supporting the mandatory vaccination compared to married/in cohabitation respondents (95% CI: 1.18–8.62). We also reported that as vaccination knowledge score increases, the probability of supporting mandatory COVID-19 vaccination increases by 1.55 times (95% CI: 1.33–1.81).

**Table 4:** Hierarchical logistic regression modeling for sociodemographic and vaccination knowledge score on mandatory vaccination support.

	**Model 1**		**Model 2**	
**Characteristics**	**OR (95% CI)**	***p*** **-value**	**OR (95% CI)**	***p*** **-value**
**Gender**				
Male	Ref		Ref	
Female	1.27 (0.81, 1.97)	0.295	1.28 (0.81, 2.03)	0.290
**Age (years)**	**1.03 (1.01, 1.06)**	**0.010**	**1.03 (1.01, 1.06)**	**0.019**
**Marital status**				
Married/In cohabitation	Ref		Ref	
Unmarried	0.72 (0.37, 1.39)	0.328	0.76 (0.38, 1.53)	0.443
Divorced/separated/widowed	1.91 (0.75, 4.90)	0.175	**3.19 (1.18, 8.62)**	**0.022**
**Underage children living in the household**				
No	Ref		Ref	
Yes	1.15 (0.73, 1.83)	0.541	1.13 (0.70, 1.85)	0.615
**Education**				
Undergraduate education	Ref		Ref	
Postgraduate education	1.34 (0.88, 2.05)	0.174	1.32 (0.84, 2.05)	0.223
**Annual income**				
Low	Ref		Ref	
Moderate	0.56 (0.07, 4.71)	0.593	1.11 (0.13, 9.67)	0.927
High	1.06 (0.13, 8.62)	0.955	1.87 (0.22, 15.76)	0.564
**Healthcare profession**				
Physicians	Ref		Ref	
Nursing staff	**0.51 (0.28, 0.93)**	**0.027**	0.64 (0.34, 1.18)	0.151
Pharmacists	0.80 (0.39, 1.64)	0.541	0.84 (0.40, 1.77)	0.657
Physiotherapists	0.65 (0.23, 1.87)	0.426	1.51 (0.48, 4.71)	0.481
Other Non-medical professionals	0.91 (0.43, 1.95)	0.810	1.58 (0.70, 3.58)	0.270
**General vaccination knowledge score (0–12)**	–	–	**1.55 (1.33, 1.81)**	**<0.001**

*OR, odds ratio; CI, Confidence interval.*

*Model 1: Sociodemographic characteristics on mandatory vaccination support (Yes vs. No).*

*Model 2: Sociodemographic characteristics and vaccination knowledge score on mandatory vaccination support (Yes vs. No).*

*Bold values indicate statistically significant associations*.

## Discussion

This study aimed to explore the factors that affect the attitudes including the role of vaccination knowledge of HCPs toward mandatory COVID-19 vaccination in Cyprus. We discovered that among the HCPs, 34.4% were in favor of mandatory COVID-19 vaccination, and 65.6% were opposed. Around half of HCPs deemed that this policy was directed against individual freedoms and violates human rights. Our findings indicate older age and general vaccination-related knowledge as the strongest predictors of mandatory vaccination support. These findings give crucial insights for tailor-made efforts by relevant health policy bodies to inform HCPs about the benefits of vaccination and address their concerns to increase vaccination uptake.

Our results revealed that approximately two-thirds of the HCPs were opposed to a mandatory COVID-19 policy. This finding of opposition to mandatory COVID-19 vaccination could be attributed to the observed decline in trust in national healthcare authorities and the government of the Republic of Cyprus during the pandemic. Also, the spread of misinformation about the efficacy and safety of COVID-19 vaccines, which has been present since the beginning of the pandemic may further contribute to this attitude. In fact, most of the HCPs have reported the expedited development and approval of the vaccine and fear of adverse side effects as the main reasons for hesitating to get vaccinated against COVID-19, whereas the internet and social media were reported as main sources of COVID-19 vaccination-related information. This agrees with previous studies underlining HCPs' doubts about the quality and procedures for the vaccine approval and fear of side effects as the main reasons for vaccine refusal (20, 33–38), while the internet and social media were identified as the primary sources of vaccination-related information in a number of studies (20, 34, 39, 40).

Similar to our results, 64.7% of healthcare workers in France were opposed to mandatory vaccination, (41) whilst around 58% of employees in health and welfare care in Germany were opposed to mandatory vaccination (42). Additionally, we reported smaller proportion of HCPs that were in favor of a mandatory COVID-19 policy, when compared to previous studies conducted among public health professionals (91%) (14) and occupational physicians in Italy (60.2%) (43), as well as among pediatricians in Turkey (59%) (44). Of interest, we discovered that around 50% of our study population agreed that HCPs should be required to get vaccinated against COVID-19. A similar agreement rate toward a mandatory COVID-19 vaccination policy for HCWs was reported in a recent study from Australia (50.4%) (45), while higher rates were reported in a recent study among Mongolian healthcare workers (96.3%) (15) and a study among medical students (58%) in the US (17). These disparities could be due to a variety of factors, including actual differences in attitudes toward mandatory vaccination between countries (i.e., cultural differences in sensitive matters such as mandates of all kinds), different data collection timeframes among studies, and baseline variability amongst the populations studied. In addition, factors such as the risk of infection, personal experience, the notion of collective responsibility, vaccination confidence, and perceptions toward infection control may also impact HCPs' attitudes toward vaccination and can also explain differences in mandatory vaccination acceptance between populations and countries (46, 47).

According to our findings, one of the strongest predictors of COVID-19 mandatory vaccination support among HCPs was older age. This is consistent with the results of recent studies among HCPs and the general population (14, 48–51). Previous studies have also shown that older age was associated with a higher intention to receive the COVID-19 vaccination among HCPs (20, 33, 36, 37). The higher probability of supporting the mandatory COVID-19 vaccination among older HCPs is not surprising, given that older age is associated with higher rates of COVID-19 mortality (52), making them more vulnerable and, thus more likely to support a mandatory COVID-19 policy in their self-interest. We also wanted to examine if the HCPs' level of vaccination knowledge influenced their opinions on whether COVID-19 vaccination should be made mandatory. Our findings revealed that a better vaccination knowledge score was associated with a higher probability of supporting mandatory COVID-19 vaccination, even after controlling for various potential confounders. Previous research has demonstrated that vaccine-related knowledge is a predictor of COVID-19 vaccination intention, implying that people are more likely to choose to be vaccinated if they have a better understanding of vaccination (17, 24, 53–55). We assume that greater health literacy and vaccine awareness may impact HCPs' attitudes and lead to a greater appreciation of the value of vaccines in combating current and future pandemics.

Herein we identified a considerably low percentage of HCPs being in favor of mandatory COVID-19 vaccination, which follows the general population's attitudes in Cyprus (51) and can be utilized by public health policymakers to understand HCPs' attitudes toward mandatory COVID-19 vaccination. In contrast with the general population, HCPs' workplace enhances the transmission of communicable diseases, especially to vulnerable individuals (56). Implementation of mandatory vaccination policies may be in line with HCPs' professional ethics to protect patients and prevent them from harm, however, could interfere with their civil rights. This study aimed purely to identify HCPs' attitudes toward mandatory COVID-19 vaccination and does not recommend the implementation of such a policy or sought to analyze legal and ethical aspects related to the vaccination mandate. Our results highlight the importance of investing in education since vaccination knowledge was associated with HCPs' attitudes toward mandatory vaccination.

Despite its originality and significance, several limitations of this study should be mentioned. Due to the study design, causal inferences cannot be made. Furthermore, because this study relied on voluntary, self-reported data, we cannot rule out additional types of bias, such as social desirability, which are common in surveys. Participants' recruitment method and data collection characterized by an online convenience sampling strategy may restrict our study's representativeness. In addition, we cannot rule out the possibility that people without access to technology are underrepresented in our sample, while certain sub-groups may be oversampled, lowering the study's overall reliability. Furthermore, we were unable to calculate the response rate for our online survey because there is no way of knowing how many individuals saw the survey or its links but chose not to participate. Also, our research examined the general vaccination knowledge rather than specialized knowledge regarding COVID-19 vaccination. It is unclear whether these two categories of knowledge vary in any way. Finally, these findings apply solely to the HCPs of Cyprus and cannot be generalized to other countries.

## Conclusions

To our knowledge, this is the first study that investigates the attitudes of HCPs toward mandatory COVID-19 vaccination and its association with general vaccination knowledge. This study found that about two-thirds of the HCPs were opposed to a mandatory COVID-19 policy. Older age and general vaccination-related knowledge were associated with mandatory vaccination support. Mandatory vaccination policies should be implemented with careful planning and consultation to avoid unintended consequences. Further studies will be required to assess the association between COVID-19 vaccination-related knowledge and attitudes toward mandatory vaccination.

## Data Availability Statement

The raw data supporting the conclusions of this article will be made available by the authors, without undue reservation.

## Ethics Statement

This study was approved by the Cyprus National Bioethics Committee (CNBC) (EEBK EΠ 2021.01.219). The participants provided their electronic informed consent to participate in this study.

## Author Contributions

KG: conceived and designed the survey, collected, analyzed the data, draft the original manuscript, interpreted the results, and supervised the process. MK: designed the survey, collected, analyzed the data, draft the original manuscript, and interpreted the results. MC and AK: interpreted the results. GF: draft the original manuscript and interpreted the results. All authors take responsibility for all aspects of the reliability and freedom from bias of the data presented and their discussed interpretation. All authors read and approved the final manuscript. All authors contributed to the article and approved the submitted version.

## Conflict of Interest

The authors declare that the research was conducted in the absence of any commercial or financial relationships that could be construed as a potential conflict of interest.

## Publisher's Note

All claims expressed in this article are solely those of the authors and do not necessarily represent those of their affiliated organizations, or those of the publisher, the editors and the reviewers. Any product that may be evaluated in this article, or claim that may be made by its manufacturer, is not guaranteed or endorsed by the publisher.
