# Supplementary material for: Mandatory COVID-19 Vaccination for Healthcare Professionals and Its Association With General Vaccination Knowledge: A Nationwide Cross-Sectional Survey in Cyprus
Source: Front Public Health. 2022 May 11;10:897526. doi: 10.3389/fpubh.2022.897526 (PMC9130732; doi:10.3389/fpubh.2022.897526)
Supplement: Supplementary file 1 [file Data_Sheet_1.ZIP › Supplementary Table 1.docx]

| **Supplementary Table 1.** Information about participants’ health status and attitudes towards healthcare services, overall and by mandatory vaccination support. | | | | |
| --- | --- | --- | --- | --- |
| **Healthcare services’ information and attitudes** | **Overall**  (N=504) | **Mandatory COVID-19 vaccination** | | |
|  |  | **No**  (N=328) | **Yes**  (N=172) | **p-value** |
| **Chronic diseases (at least one)** [N^a^ (%)] | | | | |
| No | 398 (79.6) | 266 (67.3) | 129 (32.7) | 0.072^c^ |
| Yes | 102 (20.4) | 59 (57.8) | 43 (42.2) |  |
| **Use of preventive healthcare services (e.g., annual check-up)** [N^a^ (%)] | | | | |
| Not at all | 24 (4.8) | 17 (70.8) | 7 (29.2) | 0.351^c^ |
| Little | 122 (24.4) | 86 (70.5) | 36 (29.5) |  |
| Moderate | 183 (36.6) | 108 (60.0) | 72 (40.0) |  |
| Often | 146 (29.2) | 99 (67.8) | 47 (32.2) |  |
| Very often | 25 (5.0) | 16 (64.0) | 9 (36.0) |  |
| **Trust in official guidelines and recommendations by the national healthcare authorities** [N^b^ (%)] | | | | |
| No trust | 74 (14.8) | 74 (100.0) | 0 (0.0) | **<0.001**^c^ |
| Little trust | 46 (9.2) | 44 (96.6) | 2 (4.4) |  |
| Moderate trust | 125 (25.0) | 103 (83.7) | 20 (16.3) |  |
| Strong trust | 179 (35.7) | 88 (49.2) | 91 (50.8) |  |
| Very strong trust | 77 (15.3) | 17 (22.4) | 59 (77.6) |  |
| **Satisfaction with the healthcare system** [N^b^ (%)] | | | | |
| No satisfied | 58 (11.6) | 54 (93.1) | 4 (6.9) | **<0.001**^c^ |
| Little satisfied | 75 (15.0) | 64 (85.3) | 11 (14.7) |  |
| Moderate satisfied | 226 (45.1) | 146 (65.2) | 78 (34.8) |  |
| Very satisfied | 128 (25.5) | 60 (47.2) | 67 (52.8) |  |
| Extremely satisfied | 14 (2.8) | 2 (14.3) | 12 (85.7) |  |
| **Following doctor’s instructions/Medical adherence** [N^b^ (%)] | | | | |
| Not at all | 6 (1.2) | 5 (83.3) | 1 (16.7) | **0.001**^c^ |
| Little | 11 (2.2) | 9 (81.8) | 2 (18.2) |  |
| Moderate | 73 (14.6) | 57 (79.2) | 15 (20.8) |  |
| Often | 269 (53.7) | 181 (67.8) | 86 (32.2) |  |
| Very often | 142 (28.3) | 75 (52.8) | 67 (47.2) |  |
| ^a^N=500; ^b^N=501; ^c^Differences between mandatory COVID-19 vaccination groups were tested using chi^2^ test; Bold values indicate statistically significant associations. | | | | |
