# Supplementary material for: Mandatory COVID-19 Vaccination for Healthcare Professionals and Its Association With General Vaccination Knowledge: A Nationwide Cross-Sectional Survey in Cyprus
Source: Front Public Health. 2022 May 11;10:897526. doi: 10.3389/fpubh.2022.897526 (PMC9130732; doi:10.3389/fpubh.2022.897526)
Supplement: Supplementary file 1 [file Data_Sheet_1.ZIP › Supplementary Table 2.docx]

**Supplementary Table 2.** Information about participants’ COVID-19 vaccination, overall and by mandatory vaccination support.

| **COVID-19 vaccination** | **Overall**  (N = 504) | **Mandatory COVID-19 vaccination** | | |
| --- | --- | --- | --- | --- |
|  |  | **No**  (N = 328) | **Yes**  (N = 172) | **p-value** |
| **Vaccination status** [N^a^ (%)] | | | | |
| No | 147 (29.6) | 147 (100.0) | 0 (0.0) | **<0.001^h^** |
| Yes | 350 (70.4) | 178 (51.3) | 169 (48.7) |  |
| **Number of doses** [N^b^ (%)] | | | | |
| 1 | 18 (5.1) | 14 (77.8) | 4 (22.2) | **<0.001^h^** |
| 2 | 179 (50.7) | 109 (61.6) | 68 (38.4) |  |
| 3 | 156 (44.2) | 56 (36.1) | 99 (63.9) |  |
| **Type of COVID-19 vaccine** [N^c^ (%)] | | | | |
| Pfizer | 236 (66.7) | 113 (48.5) | 120 (51.5) | **0.013^h^** |
| Moderna | 30 (8.5) | 24 (80.0) | 6 (20.0) |  |
| Astra Zeneca | 50 (14.1) | 24 (48.2) | 26 (52.0) |  |
| Johnson & Johnson | 11 (3.1) | 7 (63.6) | 4 (36.4) |  |
| Combination | 27 (7.6) | 11 (40.7) | 16 (59.3) |  |
| **Intention to receive another dose if requested** [N^d^ (%)] | | | | |
| Not at all | 40 (11.0) | 39 (97.5) | 1 (2.5) | **<0.001^h^** |
| Little | 21 (5.8) | 20 (100.0) | 0 (0.0) |  |
| Moderate | 48 (13.2) | 37 (78.7) | 10 (21.3) |  |
| A lot | 96 (26.5) | 52 (54.7) | 43 (45.3) |  |
| Very much | 158 (43.5) | 40 (25.3) | 118 (74.7) |  |
| **Belief that vaccine helped to prevent COVID-19 disease?** [N^e^ (%)] | | | | |
| Not at all | 32 (8.4) | 32 (100.0) | 0 (0.0) | **<0.001**^h^ |
| Little | 33 (8.7) | 31 (93.9) | 2 (6.1) |  |
| Moderate | 59 (15.6) | 44 (75.9) | 14 (24.1) |  |
| A lot | 144 (38.0) | 70 (49.0) | 73 (51.0) |  |
| Very much | 111 (29.3) | 27 (24.5) | 83 (75.5) |  |
| **If you have not received the COVID-19 vaccine to date, do you plan to receive it?** [N^f^ (%)] | | | | |
| I do not know | 41 (26.8) | 40 (97.6) | 1 (2.4) | **<0.001**^h^ |
| No | 103 (67.3) | 102 (99.0) | 1 (1.0) |  |
| Yes | 9 (5.9) | 6 (66.7) | 3 (33.3) |  |
| **They belong to a vulnerable group (diabetic, immunosuppressed, etc.) to whom vaccination is recommended** [N^g^ (%)] | | | | |
| I do not know | 14 (2.9) | 11 (78.6) | 3 (21.4) | **0.014**^h^ |
| No | 412 (84.8) | 278 (68.0) | 131 (32.0) |  |
| Yes | 60 (12.3) | 30 (50.0) | 30 (50.0) |  |
| ^a^N=497; ^b^N=353; ^c^N=354; ^d^N=363; ^e^N=379; ^f^N=153; ^g^N=486; ^h^Differences between mandatory COVID-19 vaccination groups were tested using chi^2^ test; Bold values indicate statistically significant associations. | | | | |
