# Supplementary material for: Mandatory COVID-19 Vaccination for Healthcare Professionals and Its Association With General Vaccination Knowledge: A Nationwide Cross-Sectional Survey in Cyprus
Source: Front Public Health. 2022 May 11;10:897526. doi: 10.3389/fpubh.2022.897526 (PMC9130732; doi:10.3389/fpubh.2022.897526)
Supplement: Supplementary file 1 [file Data_Sheet_1.ZIP › Supplementary Table 3.docx]

| **Supplementary Table 3.** Participants’ general vaccination knowledge, overall and by mandatory vaccination support. | | | | |
| --- | --- | --- | --- | --- |
|  | **Overall**  (N = 504) | **Mandatory COVID-19 vaccination** | | |
|  |  | **No**  (N = 328) | **Yes**  (N = 172) | **p-value** |
| **The flu is caused by bacteria** [N^a^ (%)] | | | | |
| True | 100 (20.2) | 73 (73.0) | 27 (27.0) | 0.167^g^ |
| I do not know | 22 (4.4) | 15 (68.2) | 7 (31.8) |  |
| False | 373 (75.4) | 233 (63.0) | 137 (37.0) |  |
| **The main route of influenza transmission is through respiratory secretions** [N^b^ (%)] | | | | |
| True | 467 (93.6) | 300 (64.7) | 164 (35.3) | 0.191^g^ |
| I do not know | 10 (2.0) | 9 (90.0) | 1 (10.0) |  |
| False | 22 (4.4) | 16 (72.7) | 6 (27.3) |  |
| **There is a vaccine to prevent cervical cancer** [N^c^ (%)] | | | | |
| True | 479 (95.6) | 307 (64.5) | 169 (35.5) | 0.106^g^ |
| I do not know | 16 (3.2) | 14 (87.5) | 2 (12.5) |  |
| False | 6 (1.2) | 5 (83.3) | 1 (16.7) |  |
| **Some vaccines contain live but attenuated microorganisms** [N^c^ (%)] | | | | |
| True | 450 (89.8) | 294 (65.8) | 153 (34.2) | 0.589^g^ |
| I do not know | 31 (6.2) | 21 (67.7) | 10 (32.3) |  |
| False | 20 (4.0) | 11 (55.0) | 9 (45.0) |  |
| **Most vaccines contain adjuvants to enhance vaccine effectiveness** [N^d^ (%)] | | | | |
| True | 271 (54.6) | 167 (61.8) | 103 (38.2) | 0.077^g^ |
| I do not know | 200 (40.2) | 141 (71.2) | 57 (28.8) |  |
| False | 26 (5.2) | 15 (57.7) | 11 (42.3) |  |
| **The vaccine is a preventive measure** [N^e^ (%)] | | | | |
| True | 449 (90.5) | 278 (62.3) | 168 (37.7) | **<0.001**^g^ |
| I do not know | 10 (2.0) | 10 (100.0) | 0 (0.0) |  |
| False | 37 (7.5) | 33 (89.2) | 4 (10.8) |  |
| **The transmission of pathogenic microorganisms occurs only through infected individuals** [N^f^ (%)] | | | | |
| True | 413 (83.8) | 266 (64.9) | 144 (35.1) | 0.301^g^ |
| I do not know | 32 (6.5) | 18 (56.2) | 14 (43.8) |  |
| False | 48 (9.7) | 35 (72.9) | 13 (27.1) |  |
| **The measles/mumps/rubella (MMR) vaccine is associated with autism** [N^b^ (%)] | | | | |
| True | 48 (9.6) | 33 (68.7) | 15 (31.3) | **0.005**^g^ |
| I do not know | 150 (30.1) | 112 (75.2) | 37 (24.8) |  |
| False | 301 (60.3) | 179 (59.9) | 120 (40.1) |  |
| **If a person is vaccinated, the possibilities of transmitting the disease are higher** [N^c^ (%)] | | | | |
| True | 37 (7.4) | 34 (91.9) | 3 (8.1) | **<0.001**^g^ |
| I do not know | 39 (7.8) | 38 (97.4) | 1 (2.6) |  |
| False | 425 (84.8) | 254 (60.2) | 168 (39.8) |  |
| **Vaccines are unnecessary, as viruses can be treated with antibiotics** [N^d^ (%)] | | | | |
| True | 22 (4.4) | 21 (95.5) | 1 (4.5) | **0.001**^g^ |
| I do not know | 12 (2.4) | 11 (91.7) | 1 (8.3) |  |
| False | 463 (93.2) | 291 (63.3) | 169 (36.7) |  |
| **Vaccination needs to be done for diseases that have been eliminated** [N^b^ (%)] | | | | |
| True | 330 (66.2) | 196 (59.9) | 131 (40.1) | **0.002**^g^ |
| I do not know | 70 (14.0) | 55 (78.6) | 15 (21.4) |  |
| False | 99 (19.8) | 73 (73.7) | 26 (26.3) |  |
| **Vaccination increases the incidence of allergies** [N^b^ (%)] | | | | |
| True | 91 (18.2) | 80 (88.9) | 10 (11.1) | **<0.001**^g^ |
| I do not know | 131 (26.2) | 106 (80.9) | 25 (19.1) |  |
| False | 277 (55.6) | 138 (50.2) | 137 (49.8) |  |
| ^a^N=495; ^b^N=499; ^c^N=501; ^d^N=497; ^e^N=496; ^f^N=493; ^g^Differences between mandatory COVID-19 vaccination groups were tested using chi2 test; Bold values indicate statistically significant associations. | | | | |
