# Supplementary figures and images for: Mandatory COVID-19 Vaccination for Healthcare Professionals and Its Association With General Vaccination Knowledge: A Nationwide Cross-Sectional Survey in Cyprus
Source: Front Public Health. 2022 May 11;10:897526. doi: 10.3389/fpubh.2022.897526 (PMC9130732; doi:10.3389/fpubh.2022.897526)

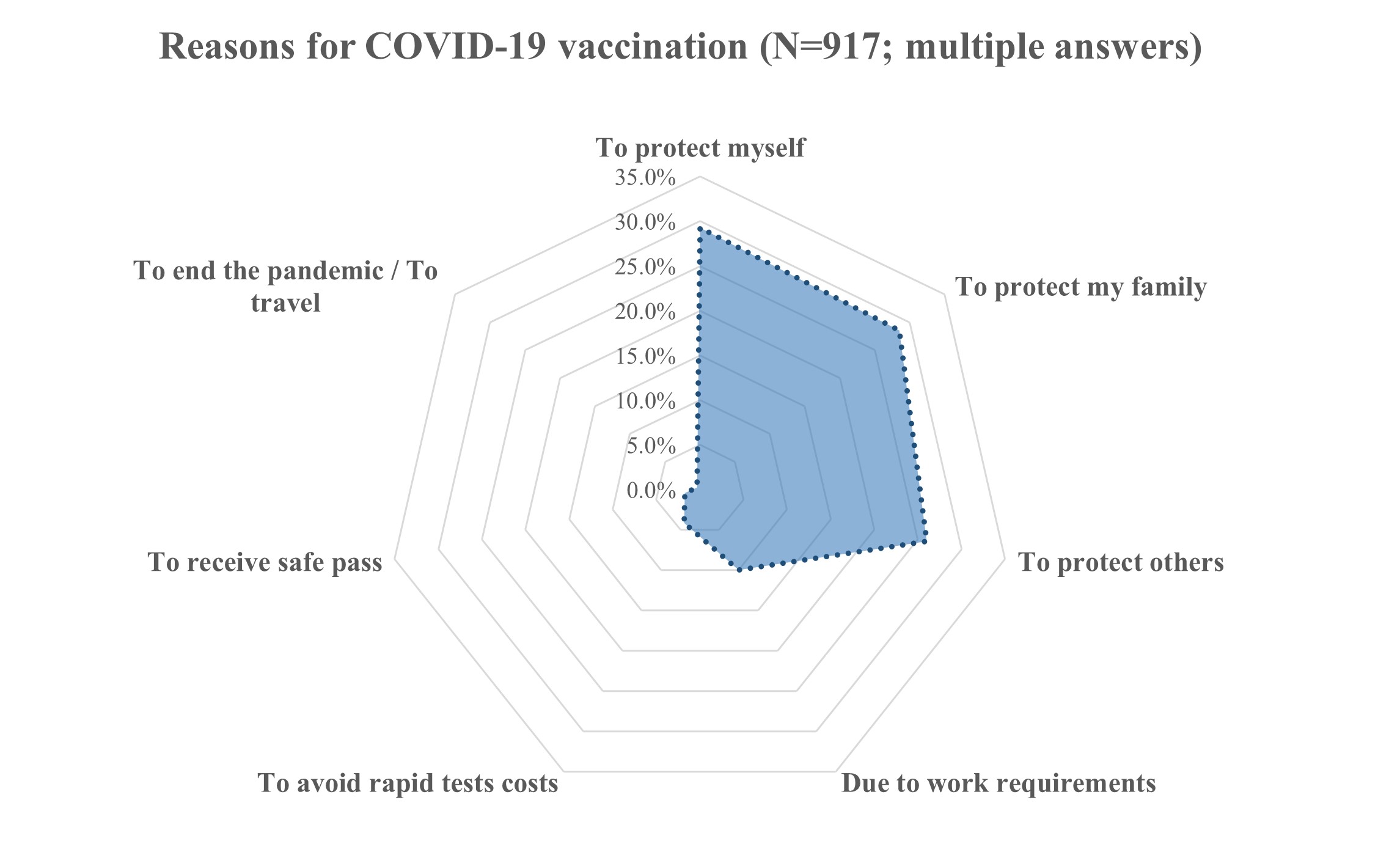

Supplement: Supplementary file 1 [file Data_Sheet_1.ZIP › Supplementary Figure 1.jpg]

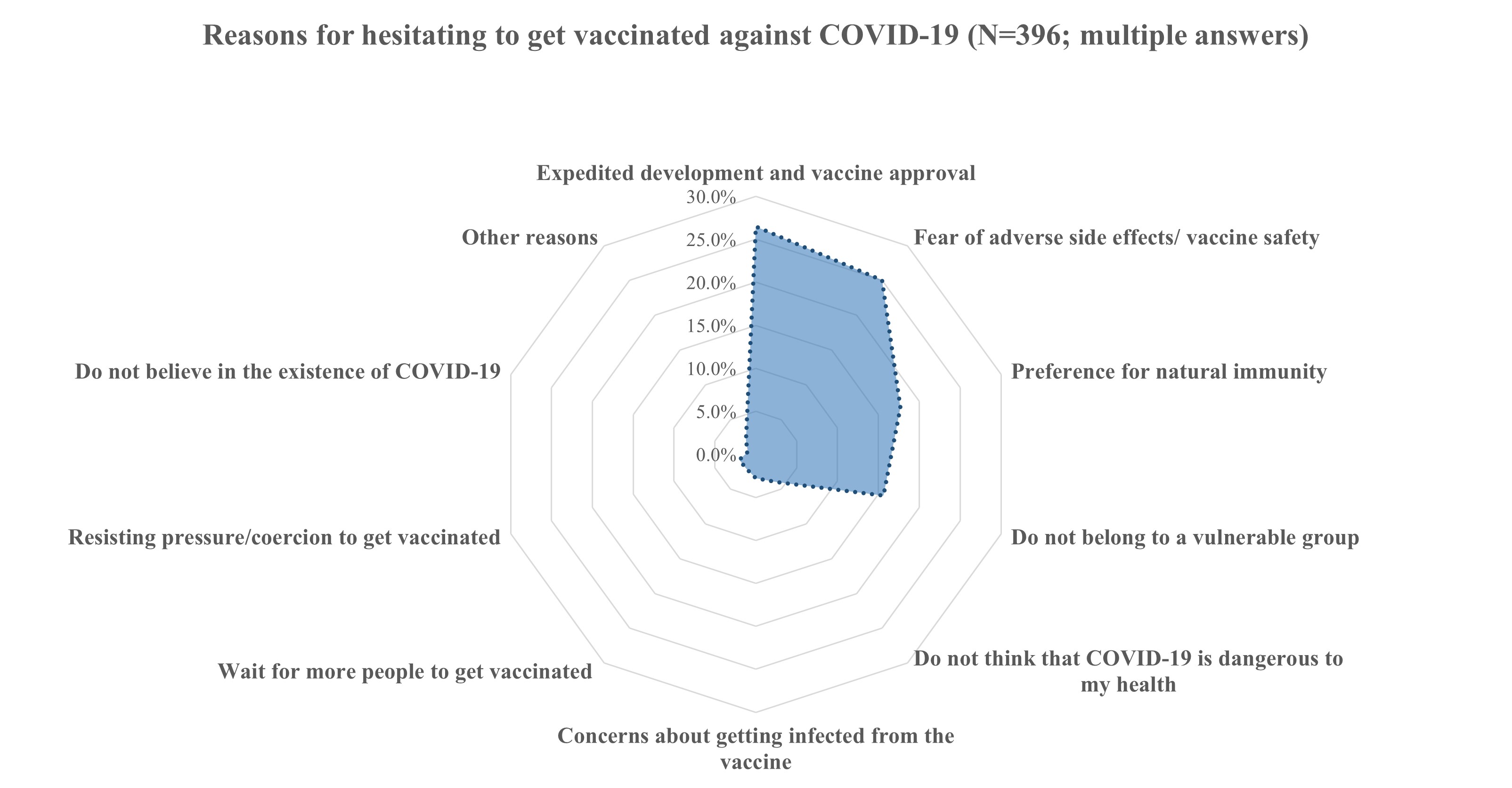

Supplement: Supplementary file 1 [file Data_Sheet_1.ZIP › Supplementary Figure 2.jpg]

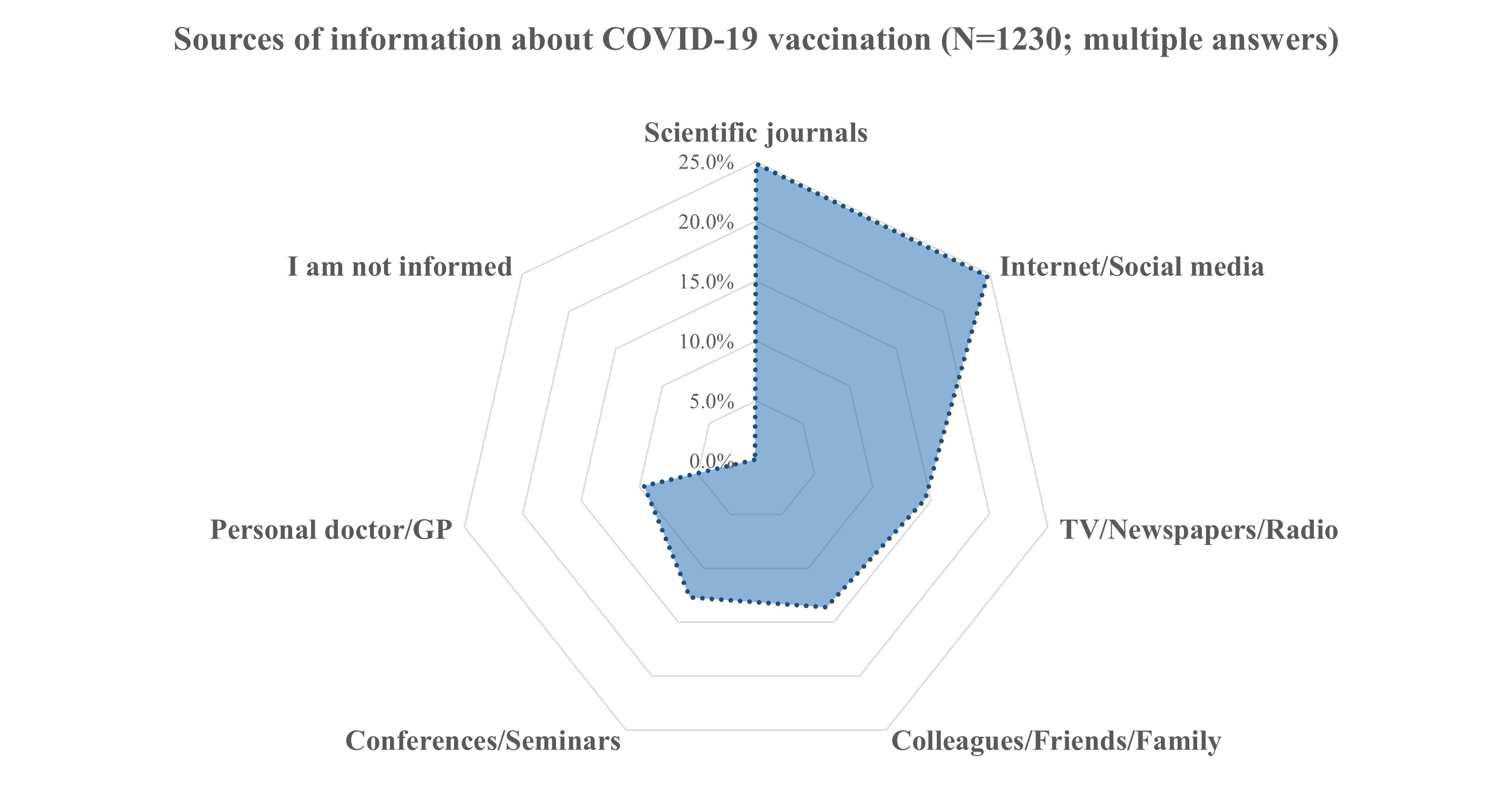

Supplement: Supplementary file 1 [file Data_Sheet_1.ZIP › Supplementary Figure 3.jpg]
